# Management of Polycaprolactone Filler‐Induced Ischemia With “THIS and FAT” Protocol: A Case Series

**DOI:** 10.1111/jocd.71116

**Published:** 2026-08-04

**Authors:** Shahriar Nazari, Mohammad Reza Pourani, Foroohe Bayatparidary, Mohammad Saleh Sadeghi, Nabil Fakih‐Gomez

**Affiliations:** ^1^ Department of ENT and Head and Neck Surgery BMI Hospital Tehran Iran; ^2^ Zibaban Clinic, Management and Research of Complications Clinic Tehran Iran; ^3^ Skin Research Center, Shohada‐e Tajrish Hospital Shahid Beheshti University of Medical Sciences Tehran Iran; ^4^ Facexpert Clinic and Academy Torremolinos Spain; ^5^ Department of Facial Plastic & Cranio‐Maxillo‐Facial Surgery Fakih Hospital Khaizaran Lebanon

**Keywords:** dermal filler, ischemia, necrosis, non–hyaluronic acid fillers, polycaprolactone

## Abstract

**Background:**

Polycaprolactone (PCL)–based fillers demonstrate clinical advantages and favorable aesthetic outcomes. However, due to the nature of their constituents, they present unique and serious challenges, particularly regarding vascular adverse events such as embolism, tissue necrosis, and visual loss.

**Aims:**

This study presents a case series of five patients with PCL filler–induced vascular complications treated using the “THIS and FAT” protocol. This protocol includes botulinum toxin, high‐dose hyaluronidase, injectable platelet‐rich fibrin (iPRF), solid platelet‐rich fibrin (sPRF), antibiotics and adjunctive medications, nanofat injection, debridement, and nanofat membrane application. The objective was to assess its clinical utility and explore potential therapeutic options in the absence of a definitive reversal agent.

**Patients and Results:**

All five patients were successfully treated with the “THIS and FAT” protocol, and no major complications were observed. The protocol provides a stepwise therapeutic strategy offering targeted interventions for each stage of ischemia.

**Conclusion:**

Managing vascular complications secondary to PCL fillers requires early recognition, rapid implementation of multimodal therapy, and a comprehensive understanding of facial anatomy. In this case series, the “THIS and FAT” protocol guided treatment through a structured, ultrasound‐assisted approach that integrates vascular, enzymatic, anti‐inflammatory, and regenerative modalities.

## 1 Introduction

Soft‐tissue filler injections are among the most frequently performed minimally invasive aesthetic procedures, with their popularity increasing significantly over the past decade. In the United States, more than 2.7 million treatments were performed in 2019, and according to the Plastic Surgery Statistics Report from the American Society of Plastic Surgeons, 7.6 million soft‐tissue filler injections were administered in 2023 by board‐certified plastic surgeons [1, 2]. While hyaluronic acid (HA) remains the most widely used agent due to its biocompatibility and reversibility, non‐hyaluronic acid (non‐HA) fillers such as calcium hydroxylapatite (CaHA), poly‐L‐lactic acid (PLLA), and polycaprolactone (PCL) have gained popularity for their longer‐lasting biostimulatory effects and structural support [3, 4].

Despite their clinical advantages, non‐HA fillers pose unique and serious challenges, particularly in the context of vascular adverse events (VAEs) such as embolism, tissue necrosis, and visual loss [5, 6, 7, 8]. Unlike HA fillers, which can be enzymatically degraded with hyaluronidase (HYAL), non‐HA fillers lack a widely accepted reversal agent, complicating the management of intravascular complications and increasing the potential for harmful outcomes [7, 9]. The first documented case of filler‐induced blindness dates back to 1906, and since then, numerous reports have confirmed that inadvertent intra‐arterial injection in high‐risk areas such as the glabella, nasal dorsum, and nasolabial folds can cause catastrophic sequelae, including irreversible vision loss within seconds [2]. Notably, non‐HA fillers are associated with more frequent and severe vascular complications compared with HA products, likely due to their physical properties and lack of degradability [10].

Among non‐HA fillers, PCL‐based products have gained prominence for their long‐lasting, collagen‐stimulating effects that can persist for several years [11, 12]. Despite their favorable safety profile, rare complications such as inflammatory nodules and ischemic events have been reported, although comprehensive clinical data remain limited [3, 11].

Recent ultrasound‐based studies have challenged the traditional view that all vascular adverse events result solely from embolism, suggesting that vasospasm may also contribute to ischemic presentations [11]. Given these risks and uncertainties, standardized management protocols for PCL filler complications are lacking, and most recommendations remain extrapolated from HA‐based treatment strategies [7, 9]. In response to this clinical gap, a study introduced the “THIS and FAT” protocol, which includes botulinum toxin (BoNT), high‐dose HYAL, injectable platelet‐rich fibrin (iPRF), solid platelet‐rich fibrin (sPRF), antibiotics and adjunctive medications, nanofat injection, debridement, and nanofat membrane application. This structured, ultrasound‐guided multimodal approach has demonstrated promising outcomes in the management of filler‐induced vascular complications [12].

As PCL fillers continue to rise in popularity, there is a critical need for systematic clinical evaluations, mechanistic investigations, and evidence‐based guidelines tailored to their unique properties. This article presents a case series of PCL filler–induced vascular complications treated using the THIS and FAT protocol to assess its clinical utility and explore potential therapeutic options in the absence of a definitive reversal agent.

## 2 Materials and Methods

### 2.1 Study Design and Patient Evaluation

A retrospective case series was conducted on five consecutive female patients who developed clinical and sonographic evidence of vascular compromise after PCL filler injection and were treated at a specialized referral center using the “THIS and FAT” protocol, including T: Botulinum *T*oxin type A (BTX‐A), H: High‐dose *H*YAL, I: *i*njectable platelet‐rich fibrin (iPRF), S: *s*olid Platelet‐Rich Fibrin (sPRF), a: *a*spirin, n: *n*anofat, d: *d*ebridement, and FAT: *fat* membrane. (Table 1) [12]. All patients provided written informed consent for treatment, data extraction, and publication of anonymized clinical images. Eligible patients presented with ischemic manifestations, including pain, livedo reticularis, cyanosis, bacterial bioburden, or early necrotic changes.

**TABLE 1:** Treatment sequence and technical details of the THIS and FAT protocol for PCL filler–induced ischemia.

Component	Preparation	Administration/dose	Timing and interval
Botulinum toxin type A	Reconstituted according to the manufacturer's sterile technique	Injected into the affected vascular territory and ischemic border as an early vascular‐modulating intervention; dose individualized according to facial site, ischemic severity, and risk of functional or cosmetic asymmetry	Administered as the first acute intervention before serial HYAL injections to reduce vasospasm and improve regional perfusion during the subsequent rescue period. Contralateral balancing may be considered for cheek or nasolabial involvement
Hyaluronidase	1500 IU diluted in 1 mL of plain 2% lidocaine to reduce pain during repeated injections and provide a suitable dilution medium	Perivascular injection into the ischemic angiosome; ultrasound‐guided intra‐arterial or periarterial bolus attempted when the involved artery was visualized. Total dose in this series: 4500–7500 IU	Repeated every 20 min as 1500‐IU aliquots. CRT and high‐frequency ultrasound were reassessed after each interval, and injections were continued until clinical perfusion improved and ultrasound confirmed arterial patency or adequate restoration of flow
iPRF	Whole blood in PET tubes without anticoagulant; centrifugation at 4000 rpm for 2 min	Injected into perilesional ischemic tissue and/or applied to the ischemic wound surface according to injury depth and extent	Initiated after vascular rescue for stage II or higher lesions; repeated during dressing changes or follow‐up visits when persistent ulceration, bacterial bioburden, or delayed epithelialization was present
sPRF	Whole blood in PET tubes without anticoagulant; centrifugation at 3500 rpm for 3 min to obtain serum platelet‐rich fibrin	Used as a solid platelet‐rich fibrin dressing over ischemic skin lesions	Applied in the wound‐care phase for stage II or higher lesions and renewed during dressing changes when clinically indicated
Debridement/dermabrasion	Conservative mechanical debridement with soft dermabrasion of nonviable surface tissue	Selective removal of necrotic tissue or bacterial bioburden to reduce bacterial load and prepare the wound bed	Used for stage III or higher ischemia, particularly when bacterial bioburden, coagulative necrosis, eschar, or nonviable tissue was present
Nanofat/fat membrane	Fat harvested using modified Klein's solution and processed by sequential filtration, with final filtration through a 500‐μm filter	Nanofat injected intradermally and intralesionally into the ischemic wound bed using a 27‐gauge needle. For fat membrane coverage, the prepared nanofat or microfat membrane was trimmed to the size and contour of the ischemic defect, placed over the debrided wound surface, and secured to surrounding viable skin with fine interrupted sutures	Reserved for advanced or post‐ischemic wounds after perfusion stabilization and wound‐bed preparation; a non‐adherent dressing was applied over the membrane to maintain contact during early healing
Adjunctive medications	Medication selected according to ischemic stage, infection risk, wound status, and contraindications	Antiplatelet or anticoagulant therapy, anti‐inflammatory medication, vasodilatory support, and antibiotics were used when clinically indicated	Typically initiated at presentation and adjusted during follow‐up; antibiotics or vasodilatory support were commonly continued for 7–10 days when indicated

At presentation, demographic data, medical and injection history, time from filler injection to referral, injected facial site, injection technique and volume when available, clinical ischemic signs, digital photographs, capillary refill time (CRT), and high‐frequency ultrasound findings were recorded. Ultrasound was used to identify the affected vascular territory, assess arterial occlusion or hypoperfusion, guide targeted injections when feasible, and confirm post‐treatment restoration of flow. Patients were monitored clinically and sonographically during the acute intervention and followed within 24 h, weekly during the first month, and monthly thereafter until complete epithelial recovery or a stable scar outcome.

### 2.2 Treatment Protocol

The treatment sequence was standardized but stage‐adapted and was organized into four components: early vascular rescue, PRF‐based wound support, wound‐bed preparation with regenerative fat‐based coverage when required, and adjunctive pharmacologic support.

#### 2.2.1 Botulinum Toxin and Hyaluronidase

In the acute rescue phase, botulinum toxin type A (Dyston, abobotulinum toxin A; Atra Zist Aray Biopharmaceutical, Iran) was administered as the first therapeutic step before serial HYAL injections. This sequence was selected to initiate early vascular modulation before repeated HYAL aliquots, with the aim of reducing vasospasm and improving regional perfusion during the subsequent rescue period. BoNT was injected into the affected vascular territory and ischemic border, with the dose individualized according to the involved facial region, ischemic severity, and risk of functional or cosmetic asymmetry.

Hyaluronidase (HYAL) was prepared as 1500 IU diluted in 1 mL of plain 2% lidocaine. Lidocaine was used to reduce pain during repeated injections and to provide a suitable dilution medium, as its mildly acidic pH remains within the reported activity range of HYAL. The solution was injected perivascularly into the ischemic angiosome; when the involved artery was visualized, an ultrasound‐guided intra‐arterial or periarterial bolus was attempted. After each 20‐min interval, CRT was reassessed and high‐frequency ultrasound was repeated to evaluate arterial patency and restoration of flow in the affected vessel. Serial HYAL injections every 20 min (consistent with CMAC protocol) were continued until clinical perfusion improved and ultrasound confirmed reopening of the involved artery or adequate restoration of arterial flow. In the present series, total HYAL dose ranged from 4500 to 7500 IU, corresponding to three to five 1500‐IU aliquots during the acute rescue session.

#### 2.2.2 PRF‐Based Wound Support

Autologous fibrin‐based therapy was then applied for wound support. Injectable platelet‐rich fibrin (iPRF) was prepared from whole blood collected in polyethylene terephthalate tubes without anticoagulant and centrifuged according to the parent protocol at 4000 rpm for 2 min. Solid platelet‐rich fibrin (sPRF) was prepared under similar conditions at 3500 rpm for 3 min. iPRF was applied to the ischemic wound surface and/or injected into the perilesional ischemic tissue according to the depth and extent of injury, while sPRF was used as a biologic dressing over stage II or higher ischemic skin lesions. PRF‐based treatment was initiated during the early wound‐care phase after vascular rescue and repeated at dressing changes or follow‐up visits when persistent ischemic ulceration, bacterial bioburden, or delayed epithelialization was present.

#### 2.2.3 Debridement, Nanofat, and Fat Membrane

In patients with bacterial bioburden, coagulative necrosis, eschar formation, or nonviable tissue, selective debridement and soft dermabrasion were performed to reduce bacterial load and prepare the wound bed. For advanced or post‐ischemic wounds, nanofat was used as a regenerative adjunct. Fat harvesting was performed under local anesthesia using modified Klein's solution composed of 500 mL normal saline, 10 mL 2% lidocaine, and 1 mL of 1:1000 adrenaline. Lipoaspirate was harvested under low negative pressure with a microfat cannula attached to a 10‐mL Luer‐Lock syringe. Nanofat was produced by emulsifying microfat through sequential filtration, with final filtration through a 600‐μm filter. The final nanofat was injected intradermally and intralesionally into the ischemic wound bed using a 27‐gauge needle.

Fat membrane (combination of nanofat/microfat and PRF with 1:1 ratio) coverage was performed by trimming the prepared nanofat/microfat membrane according to the size and contour of the ischemic defect, placing it directly over the debrided wound surface to cover the exposed ischemic area, and securing it to the surrounding viable skin with fine interrupted sutures [13]. A non‐adherent dressing was then applied over the membrane to maintain contact with the wound bed during the early healing phase.

#### 2.2.4 Adjunctive Medications and Outcomes

Adjunctive medications were individualized according to ischemic severity and infection risk and included antiplatelet or anticoagulant therapy, anti‐inflammatory medication, vasodilatory support, and antibiotics when bacterial bioburden or ulceration was present. The parent protocol commonly used aspirin 100 mg daily, pentoxifylline 400 mg three times daily, clindamycin 150 mg three times daily, and levofloxacin 750 mg daily for 7–10 days; in the present PCL series, medication selection was adapted to patient‐specific risks, contraindications, and wound status.

Primary outcomes were ultrasound‐confirmed restoration of arterial flow and clinical resolution of ischemic signs. Secondary outcomes included time to improvement, complete epithelial recovery, scarring, infection, functional deficit, need for debridement or regenerative procedures, and treatment‐related adverse events.

## 3 Results

### 3.1 Case 1

An 18‐year‐old female presented with bacterial bioburden, livedo reticularis, and focal areas of coagulative necrosis with eschar formation. She arrived 62 h after receiving PCL filler. She reported immediate bleeding and sudden pain following the injection, which had been placed in the soft triangle. Her medical history included nasal reconstruction surgery 1 year earlier and multiple subsequent nasal filler injections for refinement of the surgical outcome. Ultrasound examination revealed complete occlusion of the lateral nasal artery. The “THIS AND FAT” protocol was initiated promptly, resulting in gradual symptom resolution, and arterial flow was restored after the administration of 6000 IU of HYAL.

### 3.2 Case 2

A 56‐year‐old female presented 90 h after PCL filler injection into the nasolabial fold (NLF). She experienced immediate pain followed by nasal bleeding several hours later. She presented with ischemic involvement of the NLF and nasal ala. She was treated with the “THIS AND FAT” protocol, and following the injection of 6000 IU of HYAL, her symptoms improved significantly. Ultrasound confirmed restoration of lateral nasal artery flow (Figure 1).

**FIGURE 1:**
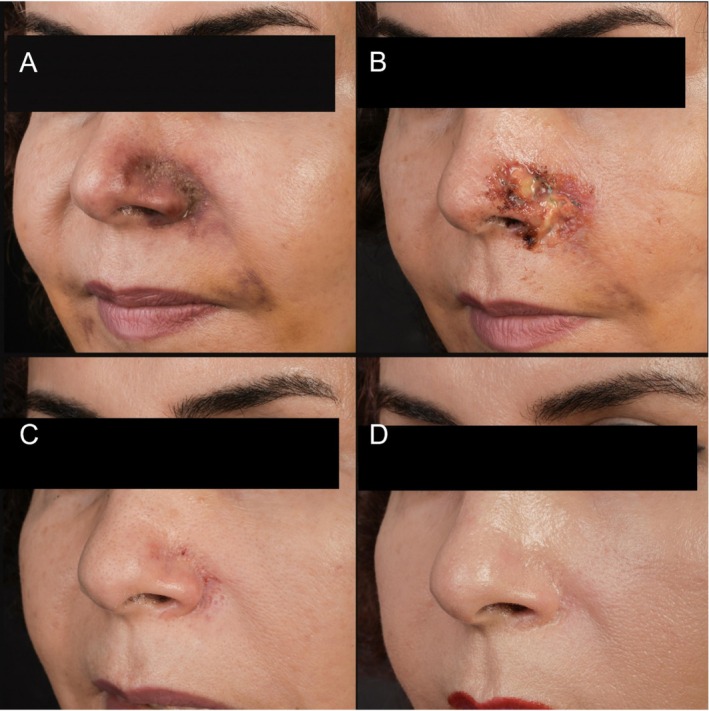
(A) Clinical presentation of a 56‐year‐old female with ischemia 90 h after PCL filler injection; (B) Appearance 1 week after receiving the “THIS and FAT” treatment; (C) Significant improvement of ischemia 1 month after treatment; (D) Complete resolution of the ischemic zone one and a half months after the “THIS and FAT” protocol.

### 3.3 Case 3

A 32‐year‐old female was referred after receiving 0.05 cc of PCL filler for acne scar treatment. She developed cyanotic discoloration shortly after injection. Sonographic imaging revealed occlusion of the supratrochlear artery. The “THIS AND FAT” protocol was started immediately, and following 4500 IU of HYAL, arterial flow returned to normal.

### 3.4 Case 4

A 32‐year‐old female presented with livedo reticularis and bacterial bioburden 90 h after receiving 1 cc of PCL filler to the cheeks using a 25‐G cannula. She was promptly treated with the “THIS AND FAT” protocol. After administration of 7500 IU of HYAL, she reported marked symptomatic improvement, and ultrasound confirmed restoration of transverse facial artery flow.

### 3.5 Case 5

A 22‐year‐old female was referred 114 h after PCL filler injection. She presented with livedo reticularis and pustular lesions on the right cheek. She had undergone subcision and PCL injection for acne scars using a cannula. Sonographic assessment revealed occlusion of the transverse facial artery. The “THIS AND FAT” protocol was initiated, and after 7500 IU of HYAL, arterial flow was restored with gradual clinical improvement (Figure 2).

**FIGURE 2:**
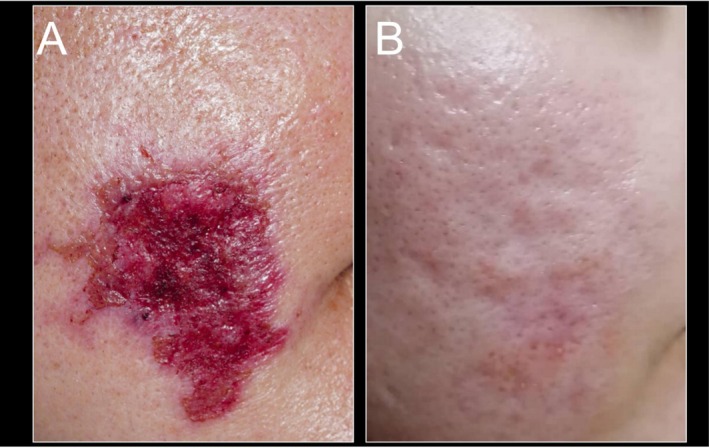
(A) Clinical presentation of a 22‐year‐old female who developed ischemia 114 h after receiving PCL filler; (B) Substantial improvement observed 10 days after treatment with the “THIS and FAT” protocol.

Five female patients (ages 18–56 years) presented with vascular complications following PCL filler injections. The interval from PCL filler injection to referral ranged from 62 to 114 h. All patients exhibited hallmark signs of vascular compromise, including livedo reticularis, cyanosis, pain, bacterial bioburden, or early necrosis, and each underwent high‐frequency ultrasound evaluation confirming arterial occlusion in vessels such as the lateral nasal artery, supratrochlear artery, and transverse facial artery. All cases were treated using the “THIS and FAT” protocol, with HYAL doses ranging from 4500 to 7500 IU, alongside BoNT, anti‐inflammatory medications, anticoagulants, and regenerative therapies (iPRF, sPRF, nanofat, or nanofat membrane) tailored to ischemic severity.

Restoration of arterial flow was achieved in all five patients, as confirmed by post‐treatment ultrasound. Clinical symptoms, including pain, discoloration, and ischemic skin changes, improved progressively, with substantial recovery observed within 7–14 days. No patient developed full‐thickness tissue loss requiring surgical reconstruction. Complete resolution occurred within 4–6 weeks in four cases, while one patient with more extensive involvement required approximately 6 weeks for full healing. No secondary infection, long‐term functional deficit, or treatment‐related adverse effect was observed at final follow‐up. Overall, all patients demonstrated full clinical recovery after application of the “THIS and FAT” protocol (Figures 1 and 2).

## 4 Discussion

Despite the overall safety of dermal fillers, vascular adverse events remain among the most serious complications in aesthetic practice. This challenge is particularly important for PCL‐based fillers, which are composed of 25–50 μm polycaprolactone microspheres suspended in a carboxymethylcellulose gel carrier and provide long‐lasting collagen stimulation rather than simple temporary volumization [4, 11]. Because PCL microspheres cannot be enzymatically degraded by hyaluronidase, ischemic complications after PCL injection require early recognition and multimodal management rather than reliance on a single reversal agent [14, 15].

PCL‐related complications may range from transient inflammatory reactions to rare vascular events. Mild reactions such as edema, erythema, tenderness, bruising, and delayed nodules have been described after PCL injection [3, 4]. Although vascular compromise is uncommon, it may present with pain, livedo reticularis, cyanosis, bacterial bioburden, or early necrotic change, as observed in the present series. Previous reports have also described delayed ischemic presentations after PCL injection, including complete recovery after timely multimodal treatment and successful management of facial artery embolization using adjunctive pharmacologic therapy [14, 16]. These findings emphasize that early recognition and prompt intervention are essential, while larger studies are still needed to define the incidence, mechanisms, and optimal management of PCL‐associated ischemia.

Prevention and early recognition remain central to reducing the severity of filler‐related vascular complications. Previous recommendations have emphasized detailed anatomical knowledge, slow and low‐pressure injection, appropriate depth control, use of blunt cannulas when suitable, aspiration, and avoidance of high‐risk vascular zones [7, 9]. Ultrasound may further improve safety by allowing vascular mapping before injection and targeted assessment after suspected vascular compromise [17, 18].

In the absence of a specific enzymatic antidote for PCL, acute management is directed toward restoring perfusion and limiting progression of ischemic injury. The facial angiosome concept may help explain the variable clinical patterns observed in filler‐induced ischemia, as facial vascular territories are interconnected by choke vessels and true anastomoses [19, 20]. In addition to embolic obstruction, choke‐vessel vasospasm may contribute to hypoperfusion and extension of ischemic changes after filler injection [19]. In the present series, this concept guided the early use of BoNT as a vascular‐modulating step before serial HYAL treatment. In this setting, BoNT functions as an early vascular‐modulating step intended to reduce neurogenic vasoconstriction, relax spastic choke vessels, and improve microvascular perfusion before subsequent decompressive and permeability‐enhancing interventions are applied [12, 21]. Recent studies proposed vasodilatory effects of BoNT in treating several medical conditions such as vasospastic conditions and ischemic events. In recent findings, increased diameter of vasculatures following BoNT injections has been reported [21, 22, 23].

Following BoNT, serial HYAL administration was used as the next vascular rescue component. In PCL‐related ischemia, HYAL is used not to dissolve PCL microspheres, but as an adjunct to increase tissue permeability, reduce edema‐related compression, and potentially improve vascular tone [11, 12]. The 20‐min interval for serial HYAL administration followed the pulsed high‐dose strategy used in the parent “THIS and FAT” protocol and CMAC recommendations, emphasizing repeated dosing guided by clinical response, capillary refill time, and restoration of perfusion rather than by a fixed total dose [24, 25]. In the present PCL cases, each 20‐min reassessment included clinical evaluation and ultrasound assessment of the affected vessel, and serial HYAL injections were continued until clinical perfusion improved and ultrasound confirmed arterial patency or adequate restoration of flow. Kadouch et al. similarly suggested that HYAL may benefit non‐HA vascular adverse events through modulation of vascular tone rather than direct filler degradation [11].

Other pharmacologic measures, including corticosteroids, anticoagulants, aspirin, nitroglycerin, and low‐dose 5‐FU‐based regimens, have been reported in selected cases or as supportive therapies [4, 5, 16]. These adjunctive therapies may be considered according to clinical severity, inflammatory burden, perfusion status, and wound characteristics; however, their role in PCL‐induced ischemia remains insufficiently standardized and requires further clinical evaluation.

Within the THIS and FAT protocol, after initial vascular rescue, management is extended to wound‐bed control and post‐ischemic tissue repair, particularly in patients with bacterial bioburden, coagulative necrosis, or eschar formation. Regenerative approaches such as PRP, PRF, iPRF, and sPRF have been used to support angiogenesis, epithelialization, and tissue remodeling through platelet‐derived growth factors and cytokines [12, 26]. In this setting, fibrin‐based preparations such as iPRF and sPRF may be useful because their three‐dimensional fibrin matrix can retain biologic mediators at the wound site and allow gradual release during healing. Nanofat injection and fat membrane application were used in selected advanced or delayed‐healing wounds as regenerative adjuncts to support post‐ischemic tissue repair [12, 16].

In the present series, treatment was structured according to the principles of the THIS and FAT protocol and individualized based on ischemic severity, ultrasound findings, and wound characteristics. This multimodal, ultrasound‐guided approach integrates vascular modulation, serial HYAL administration, anti‐inflammatory and anticoagulant support, PRF‐based wound care, selective debridement, and regenerative fat‐based interventions when indicated. In the original case series of 25 patients with filler‐induced ischemia, the authors reported complete recovery without scarring in 92% of cases, suggesting the potential value of a staged treatment framework [12]. The favorable outcomes observed in the present PCL cases suggest that this protocol may provide a practical framework for PCL filler–induced ischemia, particularly when conventional HA‐based reversal strategies are not directly applicable.

### 4.1 Challenges and Future Directions

Despite increasing clinical experience with non‐HA filler complications, standardized management of PCL‐related ischemia remains limited. Experimental and clinical studies have explored reversal or rescue strategies for non‐HA fillers, but translating these findings into predictable clinical outcomes remains challenging [6, 8]. The present study is limited by its retrospective design, small sample size, absence of a control group, and individualized treatment selection according to ischemic severity and wound stage. Nevertheless, the favorable outcomes observed in this series suggest that a staged, ultrasound‐guided multimodal approach may be clinically useful in PCL filler–induced ischemia. Future studies should prospectively evaluate PCL‐specific treatment algorithms with standardized reporting of intervention timing, HYAL and BoNT dosing, PRF and nanofat application, treatment intervals, ultrasound endpoints, and long‐term scar outcomes.

## 5 Conclusion

PCL dermal fillers, while effective and long‐lasting, carry a distinct risk profile due to their physical properties and the absence of reliable reversal agents. Management of vascular complications requires early recognition, rapid initiation of multimodal therapy, and a comprehensive understanding of facial vascular anatomy. In this case series, treatment strategies were guided by the principles of the “THIS and FAT” protocol, which provided a structured, ultrasound‐guided framework integrating vascular modulation, enzymatic therapy, anti‐inflammatory support, and regenerative modalities. Future research should prioritize clinically validated reversal or rescue strategies for PCL fillers, advancement of real‐time imaging‐guided assessment, and development of standardized, PCL‐specific management algorithms to improve patient safety.

## Author Contributions

S.N., F.B., M.R.P., and N.F.‐G. conceptualized and designed the methodology for this study. S.N., F.B., M.R.P., and N.F.‐G. were responsible for the execution and treatment of patients in the study. S.N., M.R.P., M.S.S., and N.F.‐G. prepared the original draft. All authors contributed to the critical review, commentary, and revision of the original manuscript. All authors have read and approved the final manuscript.

## Funding

The authors have nothing to report.

## Disclosure

*Level of Evidence*: IV.

## Consent

All patients in this study provided written informed consent to access their graphics, extract their data for this study, and share their images. Written informed consent for the use of patient photographs has been obtained.

## Conflicts of Interest

The author N.F.‐G. is a consultant for Merz Aesthetics (Frankfurt, Germany).

## Data Availability

Data sharing not applicable—no new data generated, or the article describes entirely theoretical research.
